# QTc interval prolongation in HIV-infected patients: a case–control study by 24-hour Holter ECG recording

**DOI:** 10.1186/1471-2261-12-124

**Published:** 2012-12-23

**Authors:** Alessandra Fiorentini, Nicola Petrosillo, Angelo Di Stefano, Stefania Cicalini, Laura Borgognoni, Evangelo Boumis, Luigi Tubani, Pierangelo Chinello

**Affiliations:** 1 U.O. Geriatria, Ospedale di Montefiascone, via Donatori di sangue, Montefiascone, 01027, Italy; 2 Second Infectious Diseases Unit, National Institute for Infectious Diseases “L. Spallanzani”, via Portuense 292, Rome, 00149, Italy; 3 Dipartimento di Medicina Clinica, Policlinico, “Umberto I”, UOS Diagnostica Cardiovascolare Internistica Integrata, viale del Policlinico 155, Rome, 00161, Italy

**Keywords:** Cardiac autonomic function, HIV, Holter ECG, QTc interval

## Abstract

**Background:**

Aim of the study was to assess QTc interval by a 24-hour ECG recording in a group of HIV-infected individuals with a basal prolonged QTc. The risk factors associated with QTc prolongation and the indices of cardiovascular autonomic control were also evaluated.

**Methods:**

A case–control study was performed using as cases 32 HIV-infected patients with prolonged (>440 msec) QTc interval as assessed by Holter ECG, and as controls 64 HIV-infected subjects with normal QTc interval. Autonomic function was evaluated by heart rate variability analysis during 24-hour recording.

**Results:**

Duration of HIV disease was significantly longer among cases than among controls (p=0.04). Waist/hip ratio was also higher among cases than among controls (p=0.05). Frequency domain analysis showed the absence of physiologic decrease of low frequency (LF) in the night period in both cases and controls. The LF night in cases showed a statistically significant reduction when compared with controls (p=0.007).

**Conclusions:**

In our study group, QTc interval prolongation was associated with a longer duration of HIV infection and with a greater waist/hip ratio. HIV patients with QTc interval prolongation and with a longer duration of HIV infection were more likely to have an impairment of parasympathetic and sympathetic cardiac component.

## Background

QTc interval prolongation is an electrocardiographic abnormality that may cause severe arrhythmias including *torsades de pointes* and ventricular fibrillation. An increased prevalence of QTc interval prolongation and a longer QTc interval have been described among HIV-infected patients when compared to HIV-negative subjects [1-4]. QTc interval prolongation in HIV-positive patients has been associated with several drugs [5-14], with autonomic dysfunction due to HIV-associated neuropathy [15,16], and with HCV coinfection [3]. The duration of ventricular repolarization can change during the day [17-19]; aim of the study was to assess QTc interval by a 24-hour ECG recording in a group of HIV-infected individuals who presented a basal prolonged QTc. The risk factors associated with QTc prolongation and the indices of cardiovascular autonomic control were also evaluated.

## Methods

In the first phase of the study, 27 consecutive HIV-infected patients with known prolonged QTc interval (group I) as assessed by standard ECG recording, and 54 HIV-positive patients with normal QTc interval (group II) matched 1:2 with group I individuals by gender and age (± 5 years) underwent 24-hour Holter ECG recording. QTc interval assessed by standard ECG recording was defined as prolonged when >440 msec [20,21].

**Figure 1:**
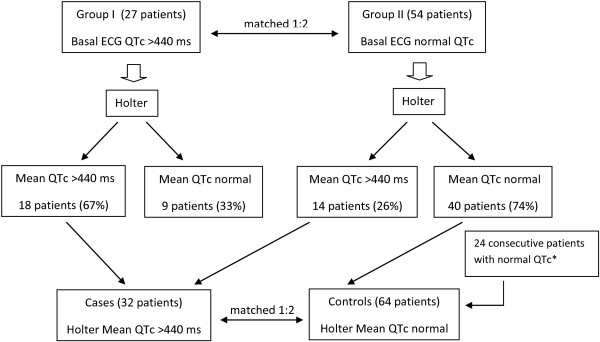
Algorithm of the study.

In the second phase of the study, a “nested” case–control study was performed using as cases the 32 patients of both group I and II with prolonged QTc interval as assessed by Holter ECG, and as controls (1:2) 64 HIV infected subjects with normal QTc interval as assessed by standard and Holter ECG, matched by gender and age (± 5 years). Forty patients came from the original group II, and 24 were consecutive HIV-infected patients with normal QTc interval at standard and Holter ECG recording. The algorithm of the study is illustrated in Figure 1. QTc interval assessed by Holter ECG recording was defined as prolonged when its mean value was >440 msec.

For each subject the following data were ascertained: demographic features; risk factors for HIV infection; HIV clinical group according to the Centers for Disease Control and Prevention (CDC), Atlanta, classification; ongoing therapy and prophylaxis; excessive alcohol intake; cardiologic risk factors including diabetes, hypertension, dyslipidemia, smoking habits, and family history; CD4+ lymphocytes count, HIV viraemia and the results of the main haematochemical tests including glycaemia, serum electrolytes, total cholesterol, high-density lipoprotein-cholesterol, low-density lipoprotein-cholesterol, and triglycerides. Hypertension was defined as a blood pressure ≥140 mmHg systolic or ≥90 mmHg diastolic or current use of antihypertensive medication [22]. Hypercholesterolaemia was defined as a serum total cholesterol level ≥200 mg/dL, hypertriglyceridaemia as a serum triglyceride level ≥200 mg/dL, and diabetes as fasting blood glucose of 126 mg/dL or greater [22]. Obesity was defined as a body mass index >30 kg/m^2^. Excessive alcohol intake was defined as follows: for women and persons >65 years of age, >7 standard drinks per week or >3 drinks per occasion; for men ≤65 years of age, >14 standard drinks per week or >4 drinks per occasion; a standard drink is approximately 12 to 14 g of ethanol [23].

All subjects, during ECG Holter registration, spent on average 8 h in bed, from 11 p.m. to 7 a.m. ECG Holter was analysed for 24 hours, at 10 minutes interval. Artificial data and arrhythmic events were excluded. Autonomic nervous system function was evaluated by heart rate variability (HRV) analysis during 24-hour ECG recording. All Holter recordings were performed using a three-channel recorder. Cardiovascular variability was analysed following the recommendations of the Task Force of the European Society of Cardiology and the North American Society of Pacing and Electrophysiology [24].

Spectral estimated of R-R interval were obtained from stationary regions free of ectopic beats and technical artifacts. The standard deviation of normal-to-normal RR intervals [SDNN (ms), correlated with total autonomic activity] and the square root of the mean of the sum of the squares of differences between adjacent NN intervals [RMS-SD (ms), correlated with parasympathetic system] were calculated and were divided in two period: night (0 am – 6 am) and day (7 am – 11 pm). Fast Fourier Transform was used to obtain power spectral estimates of HRV; total power in the frequency range (0–0.40 Hz) was divided into: very low frequency (VLF: < 0.04 Hz), low frequency (LF: 0.04–0.15 Hz, modulated by sympathetic system), and high frequency (HF: 0.15–0.40 Hz, mediated by parasympathetic system). The integral under respective power density functions were measured and were expressed in absolute units (ms^2^/Hz). Each spectral component was presented in normalized units (n.u.) by dividing LF or HF by total power minus the very low frequency (LF or HF/total power – VLF). LF/HF ratio, considered an index of cardiac sympathetic/parasympathetic tone balance, was also calculated. We examined HRV during 24 hours (total HRV), in the night time (0 am -6 am) and in the day time (7 am -11 pm).

The program calculates the QT interval from beginning of QRS to the end of the T wave. The end of the T wave was defined as the point of maximal change in the slope as the T wave merges with the baseline. QT interval was corrected for heart rate by calculating QTc. QTc was calculated by Bazett’s equation [QTc = QT/RR^1/2^] and was controlled by at least two physicians (L.T., A.F., A.D.S, and L.B.).

Data analyses were performed by Del Mar Avionics Accuplus 363, Irvine California, USA. In ten healthy subjects reproducibility was evaluated by means of the interclass correlation coefficient (ICC) comparing baseline values with the results obtained at the fourth week ICC was more > 0.7 for HRV.

Informed consent was obtained from all participants; all the investigations were performed in accordance with the principles of the Declaration of Helsinki. The research was approved by the Ethics Committee of “L. Spallanzani” National Institute for Infectious Diseases, Rome (Italy).

Statistical analysis was performed by SPSS 13.0 (SPSS Inc., Chicago, IL, U.S.A.) for Windows XP. Correlation between discrete and continuous variables were assessed by chi-square ant t-student tests, respectively. Parametric data were expressed as means ± standard deviation. A p ≤ 0.05 was considered as statistically significant.

## Results

Twenty-seven consecutive HIV-infected patients (group I) with known prolonged QTc interval (mean 464±23 msec) assessed by ECG recording at a previous study [25] underwent 24-hour Holter ECG recording. Twenty-one of them were males, their mean age was 46.6±9.5 years, and they were HIV positive since 10.5±6.4 years. Eighteen out of 27 patients (67%) had QTc interval prolongation confirmed by Holter ECG recording, while 9/27 (33%) showed a normal QTc interval at Holter ECG.

The 54 patients of group II had a normal QTc interval (mean 406±20 msec) assessed by standard ECG; they were 42 males and 12 females, HIV positive since 8.6±6.8 years, with a mean age of 46±9.2 years. Fourty of them (74%) had a normal QTc interval confirmed by Holter ECG recording, while 14/54 (26%) showed a prolonged QTc interval at Holter ECG.

Overall, 32 patients from both group I and II had a prolonged QTc interval (mean 475±33 ms) assessed by Holter ECG recording and were considered cases. They were matched by gender and age (± 5 years) with 64 controls exhibiting a normal QTc interval assessed by Holter ECG. The main demographic and clinical features of cases and controls are described in Table 1. Neither cases nor controls complained of angina or dyspnoea. Thirteen patients among cases (41%) and 17 among controls (26%) presented ectopic ventricular beats (p=0.2); 14 patients among cases (44%) and 30 among controls (47%) presented ectopic supraventricular beats (p=0.9). Duration of HIV disease was significantly longer among cases than among controls (10.9±7.1 years vs 7.8±6.4 years; p = 0.04). Cases were more frequently alcohol abusers than controls, although at a not statistically significant level (16% vs 5%; OR 3.77, 95% C.I. 0.71-21.74; p = 0.1). Waist/hip ratio was higher among cases than among controls (0.93±0.07 vs 0.90±0.07; p = 0.05). Among cases, after a mean follow-up of 4.8±0.9 years 5 patients resulted dead and 27 were still alive; among controls, after a mean follow-up of 4.1±1.1 years 2 patients resulted dead and 62 were still alive (p=0.04).

**Table 1:** Demographic and clinical features of cases and controls

**Feature**	**Cases n=32 (%)**	**Controls n=64 (%)**	**p**
Gender M	23 (72%)	46 (72%)	0.8
Age (years)*	45.7±7.6	45±9.9	0.6
BMI ≥25	15 (47%)	22 (34%)	0.3
< 25	17 (53%)	42 (66%)	
CDC stage A+B	22 (69%)	46 (72%)	0.9
C	10 (31%)	18 (28%)	
Years since HIV diagnosis*	10.9±7.1	7.8±6.4	0.04
Risk factors for HIV infection			
IVDU	8 (25%)	10 (16%)	0.3
MSM	7 (22%)	19 (30%)	
Eterosexual	16 (50%)	35 (54%)	
other	1 (3%)	0	
Arterial hypertension	4 (12%)	13 (20%)	0.5
Current smokers	18 (56%)	36 (56%)	0.8
Diabetes	0	0	
Hypertriglyceridaemia	7 (22%)	17 (26%)	0.8
Hypercholesterolaemia	7 (22%)	18 (28%)	0.7
Alcohol abuse	5 (16%)	3 (5%)	0.1
Familiarity for IHD	10 (31%)	23 (40%)	0.8
Waist circumference (cm)*	90.6±12.5	87.0±10.0	0.16
Hip circumference (cm)*	97.3±10.4	96.0±6.9	0.5
Waist/hip ratio*	0.93±0.07	0.90±0.07	0.05
Active drug abuse	3 (9%)	3 (5%)	0.4
Methadone therapy	2 (6%)	3 (5%)	1
Prophylaxis with cotrimoxazole	3 (9%)	8 (12%)	0.7
Ongoing ART	28 (87%)	53 (83%)	0.7
Type of ART			
-PI-based	12 (37%)	26 (41%)	0.4
-NNRTI-based	13 (41%)	25 (39%)	
-3 NRTI	3 (9%)	2 (3%)	
HCV positive	8 (25%)	12 (19%)	0.6
HBsAg positive	1 (3%)	1 (2%)	1
Na (mEq/L)*	139±3.2	139±3.9	1
K (mEq/L)*	4,2±0.4	4,3±0.3	0.2
Ca (mg/dL)*	9,2±0.6	9,1±0.5	0.4
CD4+ lymphocytes/mmc*	501±214	490±264	0.8
HIVRNA (cp/mL)			
<50	23 (72%)	45 (70%)	0.9
≥50	9 (28%)	19 (30%)	
QTc interval (ms)*	475±33	401±23	<0.0001
Ectopic ventricular beats	13 (41%)	17 (26%)	0.2
Ectopic supraventricular beats	14 (44%)	30 (47%)	0.9

*: mean±SD; BMI: body mass index; IVDU: intravenous drug users; MSM: men having sex with men; IHD: ischaemic heart disease; ART: antiretroviral therapy; PI: protease inhibitors; NNRTI: non-nucleoside reverse transcriptase inhibitors; NRTI: nucleoside reverse transcriptase inhibitors.

Antiretroviral drugs were not associated with QTc prolongation at a statistically significant level (Table 2). However, patients treated with zidovudine (AZT) presented QTc prolongation more frequently than patients not treated with AZT (34% vs 19%; OR 2.27 and 95% C.I. 0.78-6.62; p=0.15). Moreover, patients receiving emtricitabine (FTC) were less likely to have a QTc prolongation than patients not receiving FTC (12% vs 31%; OR 0.31 and 95% C.I. 0.08-1.12; p=0.08).

**Table 2:** Antiretroviral drugs and QTc prolongation

**Drug**	**Cases (n=32)**	**Controls (n=64)**	**p**
Zidovudine			
Yes	11	12	0.15
No	21	52	
Lamivudine			
Yes	17	28	0.5
No	15	36	
Tenofovir			
Yes	15	35	0.6
No	17	29	
Emtricitabine			
Yes	4	20	0.08
No	28	44	
Emtricitabine+tenofovir			
Yes	4	20	0.08
No	28	44	
Zidovudine+lamivudine			
Yes	7	11	0.8
No	25	53	
Didanosine			
Yes	4	4	0.4
No	28	60	
Abacavir			
Yes	3	4	0.6
No	29	60	
Efavirenz			
Yes	11	20	0.9
No	21	44	
Nevirapine			
Yes	3	6	1
No	29	58	
Atazanavir			
Yes	6	16	0.6
No	26	48	
Fosamprenavir			
Yes	2	2	0.6
No	30	62	
Lopinavir/ritonavir			
Yes	2	5	1
No	30	59	

Table 3 illustrates the indices of cardiovascular autonomic control among cases and controls. Time domain analysis showed a reduction of total SDNN in cases and controls versus normal value, without statistically significant differences between the two groups (p=0.320). Frequency domain analysis showed the absence of physiologic decreased of LF in the night period in both cases and controls. The LF night in cases showed a statistically significant reduction when compared with controls (p=0.007).

**Table 3:** Indices of cardiovascular autonomic control

	**Cases (n=32)**	**Controls (n=64)**	**p**
LF/HF ratio	2,78 ± 1,08	2,91 ± 1,23	0,306
LF (n.u.)	58, 48 ± 10,35	60,73 ± 9,68	0,151
HF (n.u.)	31,10 ± 7,43	30,72 ± 7,29	0,404
LF/HF ratio day	3,26 ±1,25	3,26 ± 1,43	0,491
LF day (n.u.)	62,91 ± 10,81	63,64 ± 10,81	0,378
HF day (n.u.)	26,85 ± 7,69	27,32 ± 7,26	0,385
LF/HF ratio night	1,90 ± 1,06	2,30 ± 1,14	0,050
LF night (n.u.)	50,30 ± 12,94	56,46 ± 10,41	0,007
HF night (n.u.)	38,76 ± 9,45	35,78 ± 9,46	0,077
SDNN (ms)	119,23 ± 38,64	122,93 ± 34,92	0,320
SDNN day (ms)	87,61 ± 25,14	97,26 ± 41,00	0,114
SDNN night (ms)	90,17 ± 27,66	104,22 ± 30,52	0,016
RMSSD (ms)	37,45 ± 14,74	40,49 ± 16,22	0,190
RMSSD day (ms)	36,16 ± 18,25	37,49 ± 16,00	0,358
RMSSD night (ms)	41,05 ± 18,00	43,23 ± 20,38	0,306

Results are expressed as mean ± SD for each variable. LF: low frequency; HF: high frequency; LF/HF: low frequency/high frequency; n.u.: normalized units; SDNN: standard deviation of all sinus rhythm RR intervals; RMSSD: the square root of the mean of the sum of the squares of differences between adjacent NN intervals; night time: 0–6 am, day time: 7 am–11 pm.

## Discussion

One third of our HIV-infected patients with known QTc prolongation measured by standard ECG recording showed a normal QTc interval when assessed by Holter ECG. Conversely, one fourth of our HIV-infected patients with a normal QTc interval measured by standard ECG had a prolonged QTc interval at Holter ECG. Beat-to-beat QTc interval measurements from 24-hour ECG recordings may provide additional information since cardiac autonomic nervous activity can modulate the variations of ventricular repolarization [18]. Data on variations of ventricular repolarization over time among HIV-infected patients are scanty [26].

In our patients, a significant relationship was found between duration of HIV infection and QTc interval prolongation. Such an association was previously reported by Charbit and coll [27]. The reasons for this relationship are unclear, but could reflect the influence of chronic infection or cumulative exposure to anti-HIV drugs on the heart and the autonomic nervous system [27]. Indeed, autonomic neuropathy has been associated with QTc interval prolongation in HIV-infected patients [15] and asymptomatic alterations of the sympatho-vagal balance can occur at the early stage of the disease, although they are more severe in the advanced stage of HIV infection [28,29]. Moreover, a selective autonomic neuropathy has been associated to the adipose redistribution syndrome in HIV-infected patients [30]. The increased waist/hip ratio observed in our HIV-positive patients with QTc prolongation could suggest a link between autonomic dysfunction, lipodystrophy, and QTc prolongation.

In our study population, antiretroviral drugs were not significantly associated with QTc interval prolongation. However, patients receiving AZT had an increased risk of QTc interval prolongation (OR 2.27), although at a not statistically significant level. AZT has been shown to prolong the QT interval in rats by activating reactive oxygen species formation in the heart mitochondria [31]. Conversely, patients receiving FTC were less likely to have a QTc interval prolongation (OR 0.31); we did not find any work in the literature reporting an association between FTC and QTc interval alteration. The limited sample size could have influenced the lack of statistical significance in our findings.

Our group of HIV-infected patients with prolonged QTc showed at follow-up an increased risk of death when compared with patients with normal QTc (p=0.04). Interestingly, none of the patients died for heart-related diseases. Our data suggest a possible association between QTc prolongation and all-cause mortality also in HIV-infected patients, likewise previously described among other non-HIV populations [32].

The HRV analysis showed a sympathetic and parasympathetic dysfunction, as demonstrated by the reduction of global autonomic system (evaluated by SDNN value) and an impairment of parasympathetic component in night time with loss of circadian rhythms both in group of HIV-infected patients with and without QTc interval prolongation. However, in HIV-infected patients with QTc interval prolongation we observed a relative reduction in night time of LF spectral component (pseudo normalization) related to an impairment in sympathetic modulation. We could hypothesize that in our study group of HIV-infected patients with QTc interval prolongation the longer duration of HIV infection could have a significant impact not only on parasympathetic cardiac regulation but also on sympathetic cardiac component with a down regulation. We suggest that an accurate valuation of autonomic cardiac system in HIV-infected patients with a long duration of HIV infection could be advisable.

Our work presents some limitations: the number of patients in both groups is limited, and the statistical differences marginal; moreover, no echocardiographic data are provided. However, to the best of our knowledge, this seems to be the largest group of adult HIV-infected patients ever studied by 24-hour Holter ECG recording.

## Conclusion

In conclusion, in our study group of HIV-infected patients the QTc interval prolongation was assessed by 24-hour Holter ECG recording and was associated with a longer duration of HIV infection and with a greater waist/hip ratio. Further studies are needed to clarify the role of antiretroviral drugs in the development of QTc interval prolongation when assessed by Holter ECG recording. Moreover, HIV-infected patients with QTc interval prolongation and with a longer duration of HIV infection were more likely to have an impairment of parasympathetic and sympathetic cardiac component.

## Abbreviations

HIV: Human Immunodeficiency Virus; HCV: Hepatitis C Virus; HRV: Heart rate variability; SDNN: Standard deviation of normal-to-normal RR intervals; RMS-SD: Square root of the mean of the sum of the squares of differences between adjacent NN intervals; VLF: Very low frequency; LF: Low frequency; HF: High frequency; ICC: Interclass correlation coefficient; AZT: Zidovudine; FTC: Emtricitabine.

## Competing interests

NP received speaker’s honoraria from Pfizer, MSD, Gilead, Novartis, Carefusion, Johnson & Johnson, Bristol Myers Squibb, Sanofi Aventis. All other authors declare that they have no competing interests.

## Authors’ contributions

AF, NP, LT, and PC conceived of the study, participated in its design and coordination and helped to draft the manuscript. SC, EB, and PC registered the Holter-ECGs and helped to draft the manuscript. AF, ADS, LB, and LT read and interpreted the Holter-ECGs, and drafted and revised the manuscript. All authors read and approved the final manuscript.

## Pre-publication history

The pre-publication history for this paper can be accessed here:

http://www.biomedcentral.com/1471-2261/12/124/prepub
